# Synergistic sonocatalytic degradation of carbamazepine and 17α-ethinylestradiol in the presence of carbon nanotube yarn^☆^

**DOI:** 10.1016/j.ultsonch.2025.107670

**Published:** 2025-11-08

**Authors:** Jong-Soo Choi, Hak-Hyeon Kim, Lakshmi Prasanna Lingamdinne, Janardhan Reddy Koduru, Yoon-Young Chang, Se Hoon Gihm, Min Jang, Chang Min Park, Yeomin Yoon

**Affiliations:** a Department of Environmental Science & Engineering, Ewha Womans University, Seoul 03760, the Republic of Korea; b Department of Environmental Engineering, Kwangwoon University, 447-1 Wolgye-dong Nowon-gu, Seoul 01897, the Republic of Korea; c AweXome Ray Inc., Anyang, Gyeonggi-Do 14056, the Republic of Korea; d Department of Environmental Engineering, Kyungpook National University, 80 Daehak-ro, Buk-gu, Daegu 41566, the Republic of Korea

**Keywords:** Carbon nanotube yarn, Sonocatalytic degradation, Carbamazepine, 17α-ethinylestradiol, Hydroxyl radical, Sonolysis

## Abstract

Pharmaceuticals such as carbamazepine and endocrine disruptors such as 17α-ethinylestradiol are persistently present in aquatic environments and pose serious ecological and human health concerns due to their chemical stability and limited removal efficiency by conventional wastewater treatment methods. In this study, a carbon nanotube yarn catalyzed high-frequency ultrasonic system was developed to synergistically enhance the degradation of carbamazepine and 17α-ethinylestradiol. carbon nanotube yarn, which is well known for its high surface area, chemical stability, and good electrical conductivity, was applied to a 970 kHz ultrasonic reactor to amplify acoustic cavitation and promote ^•^OH generation. Kinetic analysis confirmed that the combined ultrasonic US-assisted catalytic system with carbon nanotube yarn process enhanced degradation performance, with synergistic indices of 1.39 for carbamazepine and 1.73 for 17α-ethinylestradiol, significant improvement over ultrasonic alone. Enhanced radical generation was validated using electron paramagnetic resonance spectroscopy. In addition, the ultrasonic-assisted catalytic system with carbon nanotube yarn system showed stable catalytic activity and good reusability with an efficiency decrease of less than 10 % over five consecutive treatment cycles. The excellent pharmaceutical removal performance and retention of carbon nanotube yarn under repeated ultrasonic exposure indicates good catalytic performance and durability. The main mechanistic insight shows that carbon nanotube yarn promotes oxidative degradation via radical amplification. These results highlight the significant potential of the ultrasonic-assisted catalytic system with carbon nanotube yarn system as an efficient and scalable advanced oxidation technology to remediate persistent organic contaminants in environmental water.

## 1 Introduction

The discovery of additional contaminants in aquatic ecosystems is posing a major challenge to maintaining a safe environment globally. In particular, carbamazepine (CBZ), an anticonvulsant drug [1], and 17α-ethinylestradiol (EE2), a synthetic estrogen classified as an endocrine-disrupting chemical [2], are recognized as hazardous pollutants capable of causing serious harm to aquatic ecosystems and human health even at trace concentrations. Due to their resistance to biodegradation and chemical degradation, they cannot be removed by conventional treatment methods and therefore pose a persistent ecological and health risk if left unchecked [3]. The World Health Organization and the International Organization for Standardization have yet to define specific criteria for CBZs and EE2, the accumulating evidence of ecotoxicological effects highlights the urgency of establishing appropriate environmental standards.

Conventional technologies for the treatment of recalcitrant pollutants include adsorption, biological treatment, and chemical oxidation. However, these methods have serious limitations such as low treatment efficiency at trace concentrations, generation of residual by products, and high operation costs [4]. As an alternative, there is increasing attention in sonolysis, an advanced water treatment technology that uses high-frequency US to induce cavitation [5]. This process generates highly reactive radicals under extreme temperature and pressure conditions, leading to contaminant degradation [6]. Among the various operating parameters, frequency plays an critical role in determining cavitation and radical production [7]. High-frequency US typically induces more intense cavitation than low-frequency US, which generates a greater amount of reactive radicals and thus enhances contaminant degradation [8]. The 970 kHz high-frequency US used in this study generates a high density of reactive radicals and is believed to be highly effective in the oxidative degradation of persistent compounds such as CBZ and EE2.

Recent studies have reported significant improvements in the removal efficiency of these contaminants under high-frequency US system [9,10]. Manariotis *et al.* demonstrated that polycyclic aromatic hydrocarbons, such as phenanthrene and naphthalene, can be effectively degraded using high-frequency US alone without any additional catalysts, highlighting the intrinsic oxidative potential of cavitation-induced radical [9]. Similarly, Tiehm and Neis showed that 2,3,5-trichlorophenol underwent substantial dehalogenation and detoxification when subjected to high-frequency sonolysis at 618 kHz, confirming the ability of US alone to break down halogenated organic compounds and reduce their toxicity [10]. Collectively, these studies suggest that high-frequency US provides a highly effective means of initiating radical-mediated degradation, and offers a promising, chemical-free treatment for removing organic contaminants from water.

Although US treatment alone can degrade recalcitrant contaminants to a certain extent, the introduction of a catalyst is essential to enhance radical generation and maximize the overall reaction efficiency [11]. By integrating catalytic materials with US (sonocatalysis), it is possible to both enhance radical production and prolong their reactivity, thus promoting more rapid and efficient pollutant degradation [12]. Moreover, certain catalysts exhibit excellent acoustic energy absorption and conversion capabilities, which intensify cavitation strength and create synergistic effects within the system [13]. Owing to these advantages, sonocatalysis has garnered significant attention in advanced water treatment research, with representative catalysts including metal oxides such as TiO_2_ and ZnO, activated carbon-based materials, carbon nanotubes (CNTs), and MXenes [[14], [15], [16], [17]]. The superior performance of sonocatalytic processes has been validated by numerous studies [18,19]. Al-Hamadani *et al.* reported a remarkable enhancement in the removal efficiencies of ibuprofen and sulfamethoxazole when single-walled carbon nanotubes were introduced under 1,000 kHz US, with radical production and pollutant degradation both significantly increased compared to US alone [18]. Likewise, Choi *et al.* demonstrated that TiO_2_ catalysts supported on CNT-based nanostructures exhibited substantially improved sonocatalytic degradation of rhodamine B under high-frequency US, which was attributed to enhanced cavitation effects and reactive species generation [19]. Among these catalysts, CNTs show great potential because of their large specific surface area, high electrical conductivity, and excellent chemical stability, which contribute to enhanced radical generation and strong interactions with pollutants under ultrasonic conditions [12]. Recently, carbon nanotube yarns (CNTy), formed by assembling CNTs into fibrous structures, have emerged as a promising alternative for overcoming the limitations of dispersed CNTs, such as poor dispersion and recovery, and their application in sonocatalytic systems is currently being actively explored [20].

Accordingly, the aim of this study was to develop a high-frequency US-assisted catalytic process for the removal of recalcitrant organic contaminants (CBZ and EE2) from aqueous environments and to elucidate its efficiency and reaction mechanisms through both quantitative and qualitative analyses. To achieve this, a structurally stable and recoverable catalyst, CNTy, was fabricated and applied to a 970 kHz high-frequency US system to overcome the limitations of conventional dispersed catalysts and maximize the synergy between US and catalysis. This study aims to demonstrate how the catalytic functionality and structural advantages of CNTy enhance the oxidative degradation of persistent pollutants in a sonocatalytic system, ultimately providing a foundation for the development of sustainable and practically applicable water treatment technologies. In this context, we systematically investigated the effects of key operational parameters—including initial pharmaceuticals concentration, CNTy length, stirring speed, solution pH, temperature, and ultrasound power—on the degradation performance, in order to identify optimal conditions and quantify the synergy between ultrasound and CNTy.

## 2 Materials and methods

### 2.1 Chemical reagents

Detailed information on the chemical reagents and materials used in this study is provided in the Supporting Information.

### 2.2 Synthesis of CNTy

CNTy was synthesized via thermal chemical vapor deposition [21,22]. A precursor mixture composed of acetylene, ferrocene, and thiophene was introduced into the top of a vertically heated quartz reaction chamber along with a carrier gas mixture of argon and hydrogen, while the chamber was maintained at 1,473 K – 1,573 K. Under these conditions, acetylene was thermally decomposed and carbon species were converted into CNTs through catalytic reactions on the surface of the ferrocene-derived catalysts. The CNTs grew vertically and spontaneously aggregated into yarn form through π–π interactions [23]. The formed CNTs were continuously drawn from the bottom outlet of the chamber, immersed in ethanol to enhance intertube cohesion, and wound onto a rotating spool to obtain continuous fibers. To ensure sufficient mechanical strength and structural stability under ultrasonic conditions, 49 individual CNT yarns were twisted together to form a bundled CNTy configuration.

### 2.3 Apparatus and experimental procedure

The experimental setup and operating conditions are described below, and a schematic illustration is provided in Fig. S1. A 100 mL aqueous solution containing the target pharmaceuticals (CBZ and EE2), each at initial concentration of 10.0 μM, was placed in a 250 mL open-top glass beaker, which served as the reaction vessel. The physicochemical properties of CBZ and EE2 are summarized in Table S1. The solution pH was adjusted using phosphate buffer to a final concentration of 1.0 mM, and the pH was verified using a calibrated pH meter (Orion Star A215, Thermo Fisher Scientific, USA) equipped with an Orion ROSS Ultra Triode. Under ultrasonic irradiation in the presence of dissolved gases, the aqueous solution gradually acidifies due to radical reactions and the generation of dissolved carbon dioxide, affecting decomposition performance [24]. Therefore, a buffer system is necessary to minimize this effect. Among various buffer solutions, phosphate was selected for this study because it exhibits the lowest radical scavenging activity compared to carbonate, acetate, and borate [25]. Indirect ultrasonic treatment was conducted using a double-jacketed stainless-steel reactor (150 mm × 100 mm × 200 mm) connected to a recirculating chiller (Isotemp, Thermo Fisher Scientific, USA). The ultrasonic system was operated at a power density of 100 W/L. The chiller was maintained at 283 K, resulting in a solution temperature equilibrated at 295 K inside the reactor. CNTy was cut into 5 mm segments and pretreated by stirring at 300 rpm for 30 min in a 1:1 (v/v) mixture of deionized (DI) water and MeOH (100 mL per gram of CNTy) to eliminate unexpected impurities. After pretreatment, the CNTy was thoroughly rinsed with DI water and dried overnight in an oven at 378 K. Experimental parameters included stirring speed (0–400 rpm), CNTy length (5–240 mm), solution pH (4.0, 7.0, and 10.0), solution temperature (295 K–315 K), and radical scavenger concentrations (MeOH and *t*-BuOH: 1.0–100 mM). Samples were collected at 0–20 min intervals for kinetic analysis.

Collected samples were filtered using a 0.22 μm mixed cellulose ester membrane filter (Hyundai Micro, Republic of Korea) prior to quantification. CBZ and EE2 concentrations were analyzed using a 1260 Infinity high-performance liquid chromatography (HPLC, Agilent Technologies, USA) system equipped with diode array detector (DAD) and fluorescence detector (FLD), and a Zorbax C18 column (5 μm, 4.6 mm × 150 mm). The analytical details, including the mobile phase compositions and detection wavelengths, are summarized in Table S2. Total organic carbon (TOC) concentrations were determined using a TOC analyzer (TOC-L, Shimadzu, Japan).

The degradation efficiency (%) was calculated at each time point using Eq. (1):

(1) $$\mathrm{Degradationefficiency}\left(\%\right)=\left(1-C_{t}/C_{0}\right)\times 100$$

where *C_t_* and *C_0_* represent the concentrations (μM) of pharmaceuticals at time *t* and initial time (*t* = 0), respectively. In the textual description, the concentrations of the two pharmaceuticals are denoted as *C*, [CBZ], or [EE2], depending on the context, to clearly distinguish them.

The pseudo-first-order rate constant *k_obs_* was calculated using Eq. (2) [26]:

(2) $$\ln\left(C_{t}/C_{0}\right)=-k_{\mathrm{obs}}∙t$$

where, *k_obs_* is the pseudo-first-order rate constant (min^−1^) and *t* is the reaction time (min).

The synergistic indices of the US assisted catalytic system with CNTy (US/CNTy) combination process were calculated using Eq. (3) [27]:

(3) $$\mathrm{Synergeticindex}=\frac{k_{\mathrm{obs}}\left(US/CNTy\right)}{k_{\mathrm{obs}}\left(USonly\right)+k_{\mathrm{obs}}\left(CNTyonly\right)}$$

where *k_obs_* is the degradation rate constant of the pseudo-first-order kinetic model, and the combined US/CNTy process is considered to be more effective than individual US and CNTy treatments if the synergistic index exceeds 1.0.

### 2.4 Characterization

The physicochemical properties of CNTy were characterized using various analytical techniques. N_2_ adsorption–desorption isotherms at 77 K were measured with a BELSORP-max (Microtrac BEL, Japan) instrument after degassing approximately 100 mg of sample at 423 K for 6 h under vacuum (pressure <10^−5^ mbar). The specific surface area was calculated using the linear region of the Brunauer–Emmett–Teller (BET) equation [28]. The structural defects and graphitic order of CNTy were assessed by Raman spectroscopy (RAMANtouch, Nanophoton, Japan) using a 532 nm excitation laser. The intensity ratio of the D band to the G band (*I_D_/I_G_*) was used to evaluate the degree of disorder in the carbon framework. The point of zero charge (pH_pzc_) of CNTy was determined using the pH drift method [29]. The surface morphology and elemental composition were examined using field-emission scanning electron microscopy (FE-SEM, JSM-7600F, JEOL, Japan) equipped with energy-dispersive X-ray spectroscopy (EDS). Electron paramagnetic resonance (EPR) spectroscopy was conducted to identify the reactive oxidant species using 5,5-dimethyl-1-pyrroline N-oxide (DMPO, 10 mM) as the spin-trapping agent. The measurements were performed on a JES-X310 spectrometer (JEOL, Japan) (microwave frequency, 9.42 GHz, microwave power, 1.00 mW, modulation frequency, 100 kHz; modulation amplitude, 2.00 G). To further validate the generation of ^•^OH in the US assisted processes, the formation of *p*‑hydroxybenzoic acid (*p*-HBA) was monitored using benzoic acid (BA) [30]. This method is based on the selective hydroxylation of BA by ^•^OH at the *para*-position to produce *p*-HBA, which can be quantitatively analyzed using HPLC. As *p*-HBA formation occurs only in the presence of ^•^OH and does not involve other reactive oxygen species under the tested conditions, it serves as a reliable indirect indicator of ^•^OH generation. The quantification of *p*-HBA was performed using HPLC equipped with a diode array detector. The mobile phase consisted of ACN (20 %) and 50 mM phosphoric acid (80 %) at a flow rate of 1.5 mL/min. The detection wavelength was set at 270 nm.

### 2.5 River water

River water was sampled from Jungnangcheon, a tributary of the Han River (Seoul, South Korea), in June 2025 to evaluate the sonocatalytic system under environmentally relevant conditions. The collected water was immediately transported to the laboratory and prefiltered using a nylon membrane filter (0.22 μm pore size; Advantec, Japan) to remove suspended solids and particulates. After filtration, the physicochemical properties of the water, including pH, total dissolved solid (TDS), conductivity, TOC, and major ion concentrations, were analyzed to assess the background matrix. Electrical conductivity (EC) and TDS were measured using the TRACEABLE model (VWR, USA). The filtered river water was stored at 277 K and used in all subsequent experiments without further chemical treatment to preserve its native composition. The major anions (Cl^−^, NO_3_^−^, and SO_4_^2−^) were quantified by ion chromatography (ICS-1100, Metrohm, Switzerland). The major cations (Na^+^, K^+^, Mg^2+^, and Ca^2+^) were quantified using an Inductively Coupled Plasma Optical Emission Spectrometer (ICP-OES, Avio 200, PerkinElmer, USA). The physicochemical characteristics of river water, including pH, TDS, EC, TOC, and ionic composition, are summarized in Table S3.

## 3 Results and discussion

### 3.1 Characterization results

**Fig. 1:**
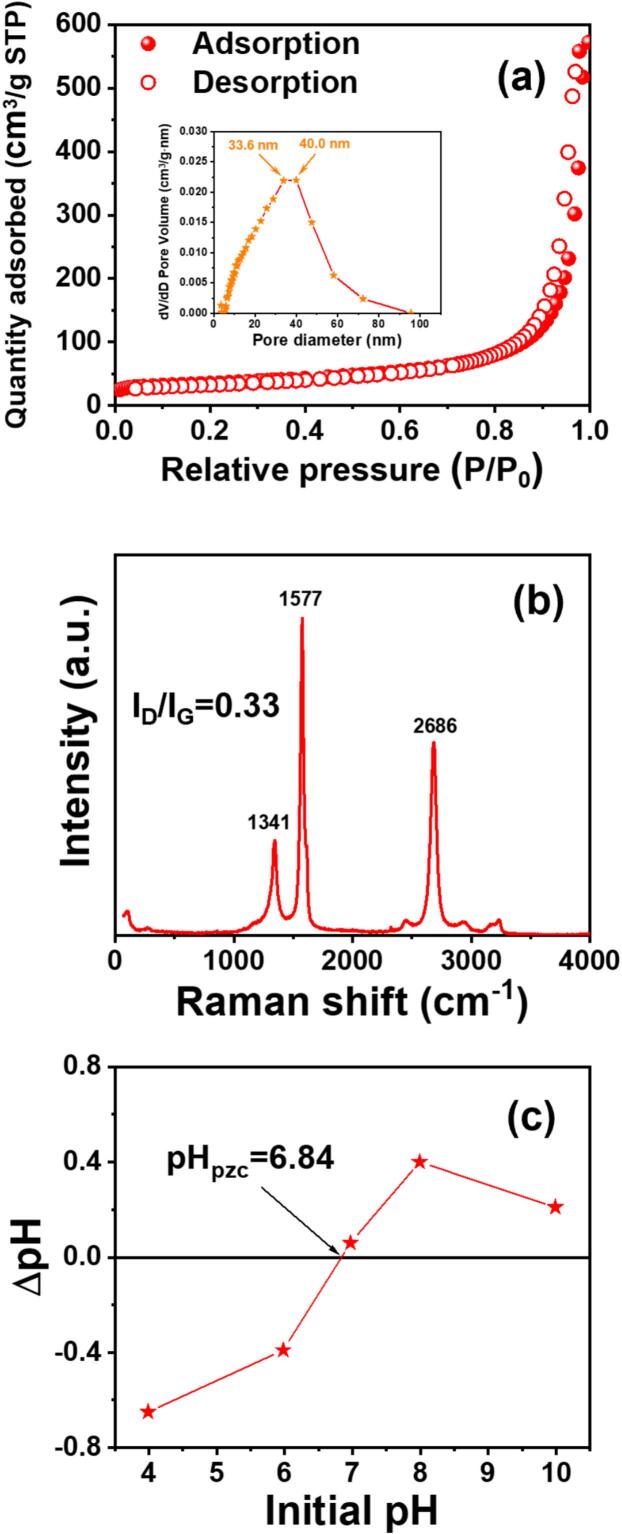
(a) Nitrogen adsorption–desorption isotherms and pore size distribution, (b) Raman spectrum, and (c) pH_pzc_ CNTy.

The physicochemical properties of the synthesized CNTy were evaluated to assess their suitability as sonocatalysts. As shown in Fig. 1a, the N_2_ adsorption–desorption isotherm of CNTy exhibited a type *IV* isotherm with an H3-type hysteresis loop, indicating a mesoporous structure [31]. The BET surface area was measured to be 128 m^2^/g, suggesting a high density of active sites for pollutant adsorption and subsequent catalytic interactions. Additionally, the pore size distribution analysis revealed that the pores are primarily distributed in the range of 3.4–95.5 nm. This meso–microporous structure facilitated the diffusion of pollutants and enhanced the interaction with reactive radicals generated under ultrasonic irradiation, thereby contributing to improved catalytic performance.

The Raman spectrum of CNTy (Fig. 1b) displays three characteristic peaks at 1,341 cm^−1^ (D band), 1,577 cm^−1^ (G band), and 2,686 cm^−1^ (2D band). The G band reflected a well-developed graphitic framework, whereas the 2D band supported the presence of ordered carbon layers [32]. Notably, the *I_D_/I_G_* ratio was calculated to be 0.30, suggesting a low degree of structural defects and a high level of crystalline ordering in the CNTy [33]. This indicates that the material consists predominantly of *sp^2^* carbon domains with minimal disorder, which facilitates efficient electron delocalization and strong π–π interactions [34].

As shown in Fig. 1c, the pH_pzc_ of CNTy was approximately 6.8, indicating that its surface is positively charged below this pH and negatively charged above it. Thus, under neutral to alkaline conditions (pH > 6.8), CNTy can promote the adsorption and degradation of cationic or neutral pollutants via electrostatic interactions.

### 3.2 Experimental results and discussion

#### 3.2.1 Effect of stirring speed

**Fig. 2:**
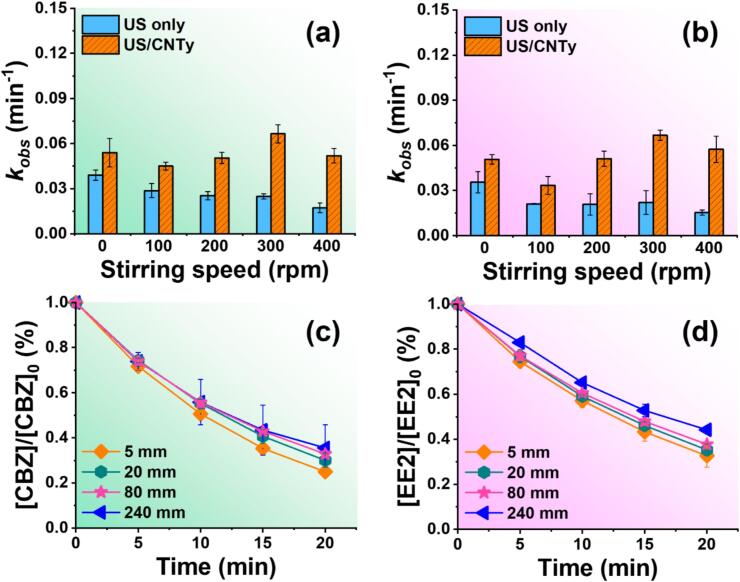
(a and b) Influence of stirring speed on the degradation rate and (c and d) effect of CNTy length on the degradation efficiency of CBZ and EE2 in US only and US/CNTy systems ([CBZ]_0_ = [EE2]_0_ = 10.0 μM, pH 7.0, 100 W/L, [CNTy] = 200 mg/L, 5 mm (a and b), 20 min, 300 rpm (c and d), and 295 K).

To investigate the influence of stirring on pollutant removal in an ultrasonic catalytic system, the effect of stirring speed on the degradation rates of CBZ and EE2 was evaluated under both ultrasonic irradiations alone (US only) and US/CNTy process. Fig. 2a and b shows that for both pollutants, degradation rates significantly improved with CNTy under ultrasonic irradiation. The lowest *k_obs_* value was observed at 100 rpm in the US/CNTy system, while a gradual improvement in *k_obs_* was observed with increasing stirring speed up to 300 rpm. The slight inhibition at 400 rpm suggests that vigorous stirring may hinder cavitation stability and suppress catalytic activity. This phenomenon highlights that the optimal stirring conditions of approximately 300 rpm enhance CNTy dispersion, maximize cavitation and promote mass-transfer synergism concurrently [35]. These trends are closely linked to the physical behavior of CNTy. CNTy tends to float under ambient conditions due to their lower density relative to water and inherently hydrophobic surface, which limits their interaction with the cavitation-induced reactive species predominantly formed in the bulk solution. At 100 rpm, insufficient mixing speed suppressed cavitation bubble formation and reduced the reaction efficiency. Optimal dispersion and interaction between CNTy and cavitation zones occurred at 200–300 rpm, which significantly accelerated the degradation rates. However, excessive agitation at 400 rpm destabilized the cavitation environment owing to surface vortex formation, negatively influencing the catalytic performance. In contrast, the US only system exhibited the highest *k_obs_* value under static conditions (0 rpm). As the stirring speed increased, a progressive decline in degradation efficiency was observed. This indicates that excessive agitation may disrupt cavitation bubble formation, thereby reducing the generation of reactive radicals that are essential for pollutant degradation [36]. Overall, these findings clearly demonstrate that stirring speed critically influences the degradation efficiency through CNTy dispersion and cavitation dynamics. Thus, optimizing the stirring speed is essential for maximizing the sonocatalytic performance.

#### 3.2.2 Effect of the CNTy length

The catalytic ultrasonic systems is often governed by the physical dispersion of the catalyst, which significantly affects its interaction with cavitation zones [35]. To investigate this, the effect of CNTy length on the degradation efficiency of CBZ and EE2 was evaluated (Fig. 2c and d). Short CNTy segments performed better than long segments due to better dispersion and minimal overlap or floatation. Long segments tend to become entangled, limiting active site exposure, reducing interaction with the ultrasonic cavitation zone and consequently reducing disintegration efficiency. These results suggest that beyond material intrinsic properties, the physical dispersion of the catalyst in the reaction medium plays an important role in determining the overall ultrasonic catalytic activity [37].

#### 3.2.3 Effect of pH

**Fig. 3:**
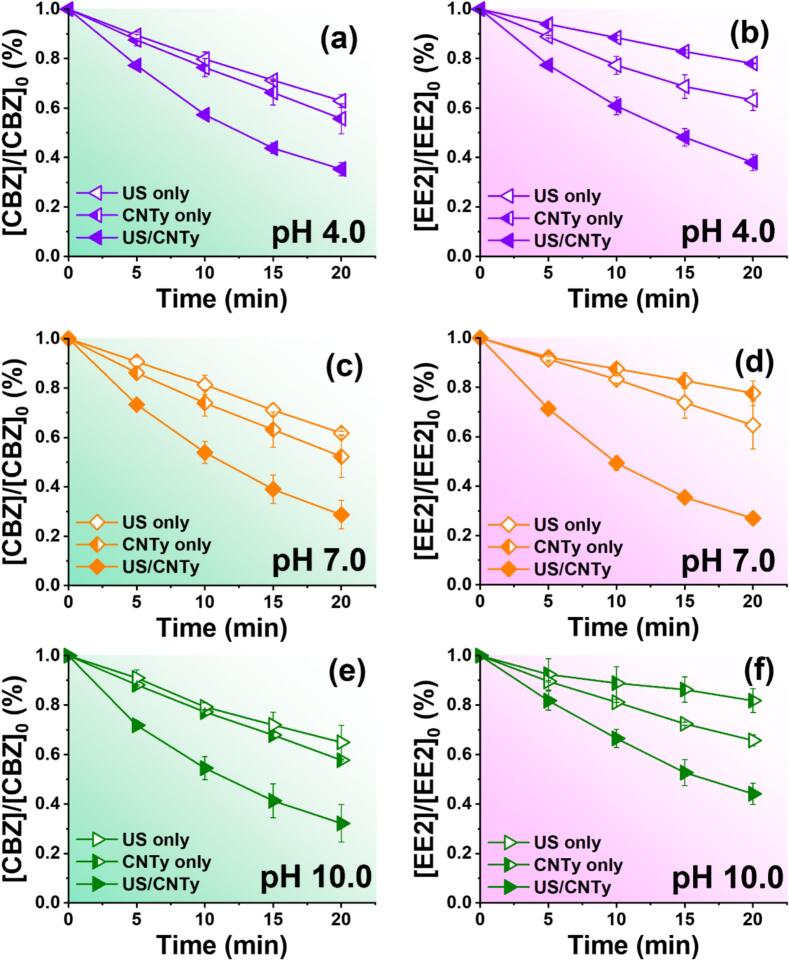
Degradation efficiency of (a, c, and e) CBZ and (b, d, and f) EE2 under different pH conditions (4.0, 7.0, and 10.0) using US only, CNTy only, and US/CNTy ([CBZ]_0_ = [EE2]_0_ = 10.0 μM, 100 W/L, [CNTy] = 200 mg/L, 5 mm, 20 min, 300 rpm, and 295 K).

To examine how pH affects contaminant degradation in ultrasonic catalytic system, CBZ and EE2 decomposition performance was evaluated at pH values of 4.0, 7.0, and 10.0 under US only, adsorption by CNTy (CNTy only), and US/CNTy systems. Fig. 3(a–c for CBZ and d–f for EE2) shows the results, with corresponding *k_obs_* values and synergy indices summarized in Table 1. In general, alkaline conditions with a predominance of OH^−^ ions enhance the generation and persistence of ^•^OH, thereby improving pollutant degradation efficiency [38]. However, the degradation efficiencies of CBZ and EE2 in the US only system showed little variation at different pH levels. The CNTy only system offers insights into pH-dependent electrostatic interactions. Based on their *pK_a_* values (CBZ: 13.9; EE2: 10.4), both compounds are predominantly neutral or weakly charged at pH 4.0 but become more negatively charged at higher pH. CNTy, with a *pH_pzc_* of 6.84, carries a positive or neutral charge below this pH and a negative charge above it. Consequently, adsorption was most favorable at pH 4.0 due to minimal electrostatic repulsion, while adsorption decreased under alkaline conditions (pH 10.0) due to surface repulsion. Notably, the US/CNTy system showed excellent degradation efficiency at all pH conditions tested, which was attributed to the interaction between ultrasonic irradiation and CNTy catalysis. Optimal performance was achieved at a neutral pH, where a balance between radical stability, effective cavitation, and favorable electrostatic interactions enhanced the catalytic activity and pollutant removal [39]. These findings emphasize the importance of pH control in improving the performance of US assisted catalytic processes. Moreover, the neutral pH conditions provide practical benefits as they match typical wastewater characteristics well and support efficient cavitation and radical-mediated degradation. Although both CBZ and EE2 remain largely neutral in the tested pH range, their interactions with CNTy differ due to structural and physicochemical properties. CBZ, with relatively lower hydrophobicity, exhibits weak adsorption onto CNTy, and its degradation is mainly governed by bulk-phase ^•^OH attack, leading to relatively stable removal across pH values. In contrast, EE2, with higher hydrophobicity and a rigid steroidal skeleton, adsorbs more strongly onto CNTy under acidic to neutral conditions, favoring surface-mediated radical reactions. At alkaline pH, reduced adsorption due to electrostatic repulsion diminishes degradation efficiency despite enhanced ^•^OH generation. These mechanistic differences explain the distinct pH-dependent degradation behaviors observed between CBZ and EE2 in the US/CNTy system.

**Table 1:** Degradation rate and synergetic index of CBZ and EE2 depending on solution pH for US only, CNTy only, and US/CNTy process.

Solution pH	Process	*k_obs_* (min^−1^)	
		CBZ	EE2
pH 4	US only	0.023	0.027
	CNTy only	0.029	0.018
	US/CNTy	0.053	0.048
	**Synergetic index**	**1.02**	**1.09**

pH 7	US only	0.024	0.022
	CNTy only	0.033	0.012
	US/CNTy	0.063	0.067
	**Synergetic index**	**1.11**	**1.94**

pH 10	US only	0.022	0.021
	CNTy only	0.027	0.009
	US/CNTy	0.057	0.042
	**Synergetic index**	**1.16**	**1.37**

#### 3.2.4 Effect of temperature

To evaluate how temperature affects radical generation and contaminant removal in ultrasonic catalytic systems [40], degradation rates were investigated at 295 K, 305 K, and 315 K under US only and US/CNTy conditions. Fig. S2a and b show the effect of solution temperature on *k_obs_* for CBZ and EE2 degradation in the US only and US/CNTy systems, respectively. For both CBZ and EE2, the degradation rate constants under the US/CNTy system improved significantly as the temperature increased from 295 K to 315 K. This trend is primarily attributed to the enhanced production and diffusion rates of reactive radicals at high temperatures and the increased frequency of collisions between radicals and polluting molecules [41]. On the other hand, it has been reported that higher temperature conditions than those performed in this study intensify the cavitation bubble formation and collapse kinetics, resulting in higher efficiency of radical generation [42]. In particular, the US/CNTy system showed a more pronounced increase in degradation performance with temperature compared to the US only system. This observation suggests that CNTy not only enhances radical generation through improved cavitation efficiency, but may also amplify non-radical mechanisms such as local thermal effects or electron transfer processes at high temperatures [11]. Thus, the combination of US and CNTy provides a robust and versatile catalytic activity, particularly at elevated temperatures.

#### 3.2.5 Effect of initial concentration

Since the initial concentration of organic pollutants significantly influences the kinetics and efficiency of sonocatalytic degradation [43], the influence of initial concentrations ranging from 2.0 to 30.0 μM on degradation efficiency was examined under CNTy alone, US alone, and combined US/CNTy conditions (Fig. S2c and d and Table 2). Notably, the US/CNTy system consistently exhibited the highest *k_obs_* values across all concentrations, confirming the synergistic effect arising from the integration of CNTy with ultrasonic irradiation. The synergistic index remained above 1.00 for both CBZ (1.35–1.43) and EE2 (1.11–1.95) at all concentrations, indicating sustained synergistic enhancement. The synergistic effect for EE2 was particularly pronounced at 10.0 μM and 30.0 μM, with synergetic indices of 1.73 and 1.95, respectively. Interestingly, the synergistic effect observed for CBZ remained relatively constant across all tested initial concentrations (2.0–30.0 μM), suggesting that CBZ degradation is primarily governed by bulk-phase radical interactions rather than CNTy surface-mediated processes [44]. In contrast, EE2 exhibited a distinct concentration-dependent synergistic enhancement, achieving notably higher synergistic indices at increased initial concentrations (e.g., 1.73 at 10.0 μM and 1.95 at 30.0 μM). This significant difference can be explained by the stronger affinity and greater hydrophobic interaction of EE2 with CNTy surfaces, resulting in increased adsorption at elevated concentrations [45]. Consequently, the EE2 molecules adsorbed onto CNTy experience enhanced proximity and interaction with surface-generated ^•^OH, thus amplifying the synergistic effect. Therefore, as pollutant concentration increased, EE2 benefited more substantially from CNTy-enhanced radical interactions compared to CBZ.

**Table 2:** Degradation rate and synergetic index of CBZ and EE2 depending on initial concentration for US only, CNTy only, and US/CNTy process.

Initial concentration (μM)	Process	*k_obs_* (min^−1^)	
		CBZ	EE2
2	US only	0.045	0.023
	CNTy only	0.014	0.015
	US/CNTy	0.084	0.043
	**Synergetic index**	**1.43**	**1.11**

10	US only	0.024	0.024
	CNTy only	0.024	0.012
	US/CNTy	0.067	0.063
	**Synergetic index**	**1.39**	**1.73**

30	US only	0.022	0.006
	CNTy only	0.016	0.007
	US/CNTy	0.052	0.025
	**Synergetic index**	**1.35**	**1.95**

#### 3.2.6 Effect of scavenger

**Fig. 4:**
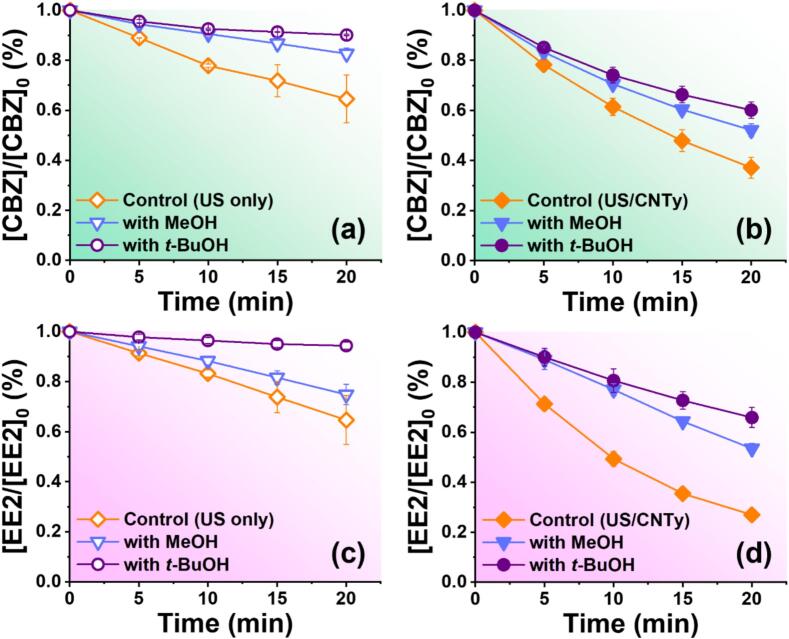
Scavenging effect of MeOH and *t*-BuOH on the degradation of (a and b) CBZ and (c and d) EE2 under US only and US/CNTy systems ([CBZ]_0_ = [EE2]_0_ = 10.0 μM, pH 7.0, 100 W/L, [CNTy] = 200 mg/L, 5 mm, 20 min, 300 rpm, 295 K, and [scavenger] = 100 mM).

Since identifying the active oxidative species is fundamental for optimizing sonocatalytic degradation of organic pollutants [46], scavenging experiments were conducted to investigate the role and significance of ^•^OH in the degradation of CBZ and EE2 under the US only and US/CNTy treatments (Fig. 4). The addition of scavengers incompletely suppressed pollutant degradation because residual removal was observed even at high scavenger concentrations (100 mM). Typically, in radical scavenging experiments, complete inhibition is expected if *^•^*OH is the exclusively active species and is completely consumed by the scavengers [47]. This partial degradation under high scavenger loading could be explained by two factors. First, this observation may suggest that other reactive species or alternative mechanisms, such as direct pyrolysis or electron transfer processes induced by high-intensity cavitation, contribute to pollutant degradation in addition to ^•^OH reactions [48]. Such alternative pathways are plausible, given the localized temperature and pressure conditions within the collapsing cavitation bubbles. Second, the spatial distribution of cavitation bubbles could play a critical role. In this study, the ultrasonic transducer was located at the bottom of the reactor, which caused radicals to be predominantly generated in the lower region. As a result, cavitation and radical formation were spatially concentrated near the transducer, leading to heterogeneous radical distribution within the reactor. This non-uniform distribution may explain the residual degradation observed even in the presence of high concentrations.

Interestingly, in the radical scavenging experiments, an unexpected observation was that *t*-BuOH exhibited a more pronounced inhibitory effect on the degradation of CBZ and EE2 than MeOH. This is contrary to the general consensus that MeOH is a more effective scavenger because of its faster reaction kinetics and ability to scavenge radicals in both bulk and surface regions [49]. The stronger inhibition effect of *t*-BuOH relative to MeOH observed in this study aligns with previous findings indicating that radicals generated by ultrasonic cavitation predominantly reside in the bulk solution rather than at catalytic surfaces [46]. This phenomenon can be explained by the rapid consumption or short lifetime of radicals generated near catalyst interfaces, limiting their effective scavenging by MeOH, whereas *t*-BuOH, being primarily reactive in bulk phases, can more effectively intercept these radicals due to its favorable reactivity profile in the bulk solution [50]. Detailed investigations of the effect of scavenger concentration revealed that the degradation of both CBZ (Fig. S3) and EE2 (Fig. S4) was inhibited in a concentration-dependent manner with increasing concentrations of MeOH and *t*-BuOH, further supporting the critical role of bulk-phase radical reactions in pharmaceutical compounds degradation.

#### 3.2.7 Reusability tests

**Fig. 5:**
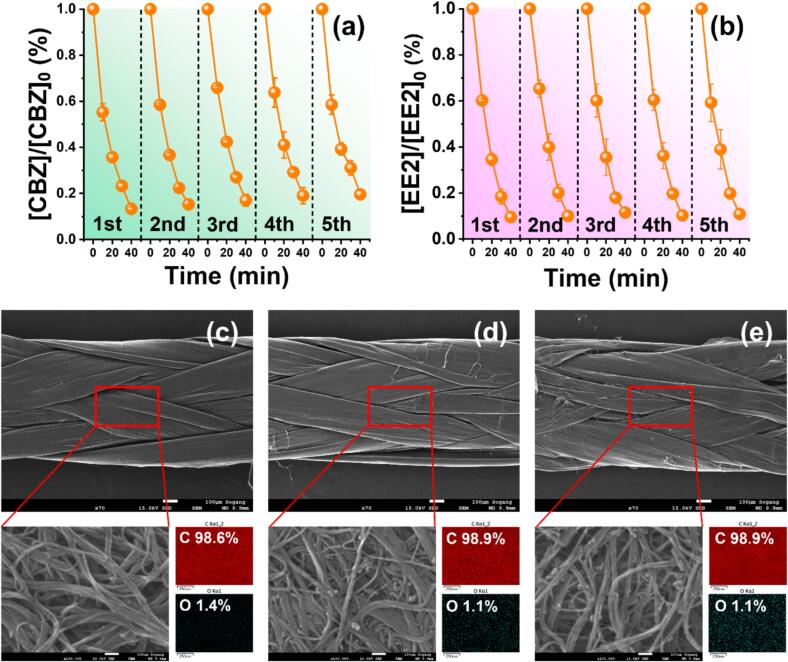
Reusability of CNTy over five consecutive treatment cycles for (a) CBZ and (b) EE2 ([CBZ]_0_ = [EE2]_0_ = 10.0 μM, pH 7.0, 100 W/L, [CNTy] = 200 mg/L, 5 mm, 40 min, 300 rpm, and 295 K). SEM images and EDS mapping of CNTy surfaces: (c) pristine CNTy, (d) CNTy after 1 h ultrasonic treatment, and (e) CNTy after five consecutive cycles.

Reusability testing is essential to assess the practical applicability of CNTy catalysts in wastewater treatment [51]. Accordingly, a preliminary experiment was conducted by extending the treatment duration to 60 min to evaluate the degradation performance of CBZ and EE2 in both the US only and US/CNTy systems, as shown in Fig. S5a and b. The US only system achieved removal efficiencies of 78.9 % for CBZ and 69.1 % for EE2 after 60 min. In contrast, the US/CNTy system exceeded 90 % removal within 40 min and reached final removal efficiencies of 95.4 % for CBZ and 97.5 % for EE2 after 60 min. Based on the above results, the stability and reusability of CNTy were evaluated over five repeated runs, each conducted for 40 min, under conditions that had previously achieved over 90 % removal efficiency for both pollutants (Fig. 5a and b). Remarkably, the degradation efficiencies remained consistently high (>80 %) across all five cycles, maintaining values of 80.4 % for CBZ and 89.1 % for EE2 even in the fifth cycle. This indicates the excellent structural stability and sustained catalytic performance of CNTy under prolonged ultrasonic exposure. Such durability likely arises from the robust structure and chemical resistance of CNTy to intense cavitation conditions. Overall, these findings demonstrate that CNTy not only enhances the efficiency of ultrasonic degradation but also exhibit superior reusability and structural stability. Unlike conventional CNT powders, which often require high temperature or energy intensive regeneration following oxidative degradation, CNT yarns maintain their robust structure under harsh ultrasonic conditions without the need for such treatments, thereby offering strong potential for continuous wastewater treatment application [20].

SEM coupled with EDS was performed before and after repeated sonocatalytic use to further investigate the structural stability of the CNTs after prolonged ultrasonic treatment. The SEM images of pristine CNTy (Fig. 5c), after 1 h of ultrasonic treatment (Fig. 5d), and after five consecutive treatment cycles (Fig. 5e) revealed only minimal structural changes. Although slight physical damage, such as partial breakage or fragmentation of individual nanotubes, was observed after prolonged exposure, the overall fibrous architecture of CNTy remained largely preserved. EDS analysis showed negligible changes in the elemental composition of all samples, confirming that the surface chemistry of CNTy remained stable throughout the treatment cycles (Fig. 5c–e). These observations clearly demonstrate that CNTy retain their structural integrity and chemical stability under intense cavitation. This robustness highlights the potential for repeated use in practical sonocatalytic wastewater treatment applications, making it a promising candidate for long-term operation.

#### 3.2.8 River water treatment

**Fig. 6:**
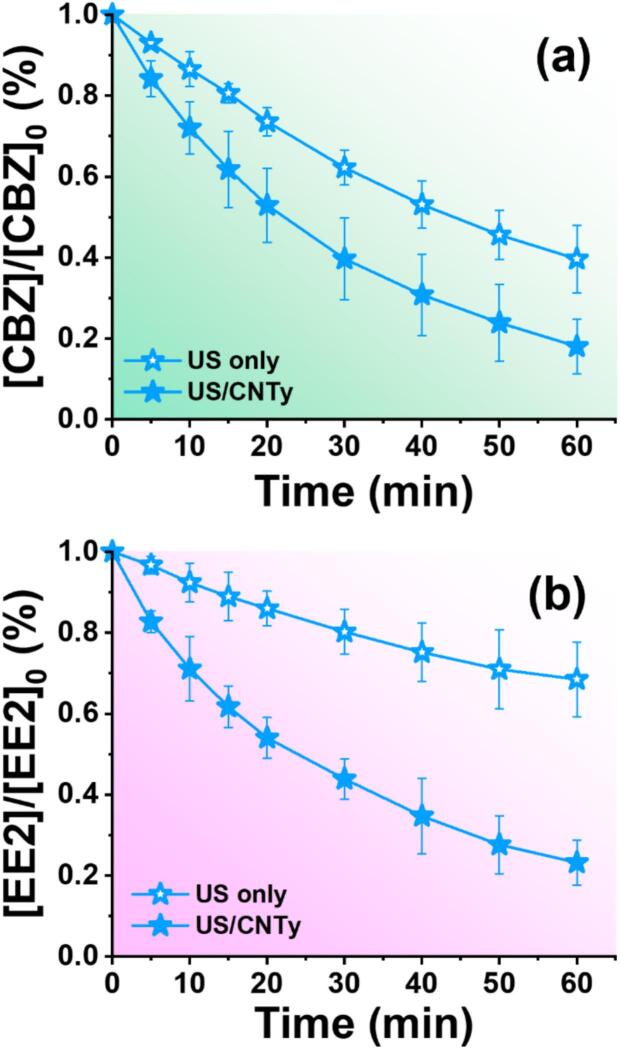
Evaluation of (a) CBZ and (b) EE2 degradation performance in river water using US only and US/CNTy systems ([CBZ]_0_ = [EE2]_0_ = 10.0 μM, 100 W/L, [CNTy] = 200 mg/L, 5 mm, 60 min, 300 rpm, and 295 K).

To evaluate the practical applicability of the US/CNTy system under environmentally relevant conditions, degradation experiments were conducted using surface water samples from the Han River (Seoul, South Korea). CBZ and EE2 were spiked into the collected river water, and the removal performance was assessed under both US only and US/CNTy conditions. The degradation performance of CBZ and EE2 in river water clearly showed that the US/CNTy system achieved significantly higher removal efficiencies than the US only system over a 60 min reaction period. For CBZ (Fig. 6a), approximately 75 % removal was observed under the US/CNTy treatment, whereas US only achieved less than 50 % removal within the same time frame. A similar trend was observed for EE2 (Fig. 6b), with US/CNTy achieving nearly 80 % degradation, in contrast to 55 % removal under US only conditions. These results are likely attributable to the influence of background organic matter present in river water on radical generation and cavitation dynamics [6,52]. When compared to the DI water background (Fig. S5), a noticeable reduction in removal efficiency was observed under the same US/CNTy conditions: after 60 min of treatment, CBZ removal decreased from 96 % (DI water) to 82 % (river water), and EE2 removal dropped from 94 % to 77 %. This performance decline suggests that naturally occurring dissolved organic matter and coexisting ions in river water may act as radical scavengers and compete for ^•^OH; in addition, high concentrations of chloride ions may narrow the effective oxidation capacity by reacting with ^•^OH to form less reactive chlorine radicals (Cl^•^ and Cl_2_^•−^), thereby suppressing pollutant degradation efficacy in natural water matrices [24,53].

#### 3.2.9 Calorimetric calculation

Energy calculation based on ultrasonic irradiation is a critical consideration for reactor design. Two approaches were considered for calorimetry. First, the expected power for a 100 mL solution was calculated through geometric scaling based on the ratio of the beaker and water bath bottom areas. Second, calorimetry was performed by monitoring the temperature rise of the water. Detailed calculations are summarized in the Supporting Information [54,55]. Geometric calculations yielded 25.7 W/L, while calorimetric calculations yielded 33.8 W/L. Compared to geometric calculations, which apply ultrasonic power limited to the output per unit area, calorimetric calculations indicated a larger energy quantity. This indicates that energy transfer, such as reflection and refraction, occurs within the ultrasonic device. Furthermore, the calorimeter calculation only considers the heat energy absorbed by the solution in the beaker, excluding factors like cavitation bubble dynamics and radical formation energy. Therefore, the actual energy affecting the interior of the beaker is estimated to exceed 33.8 W/L.

### 3.3 Key degradation mechanisms

**Fig. 7:**
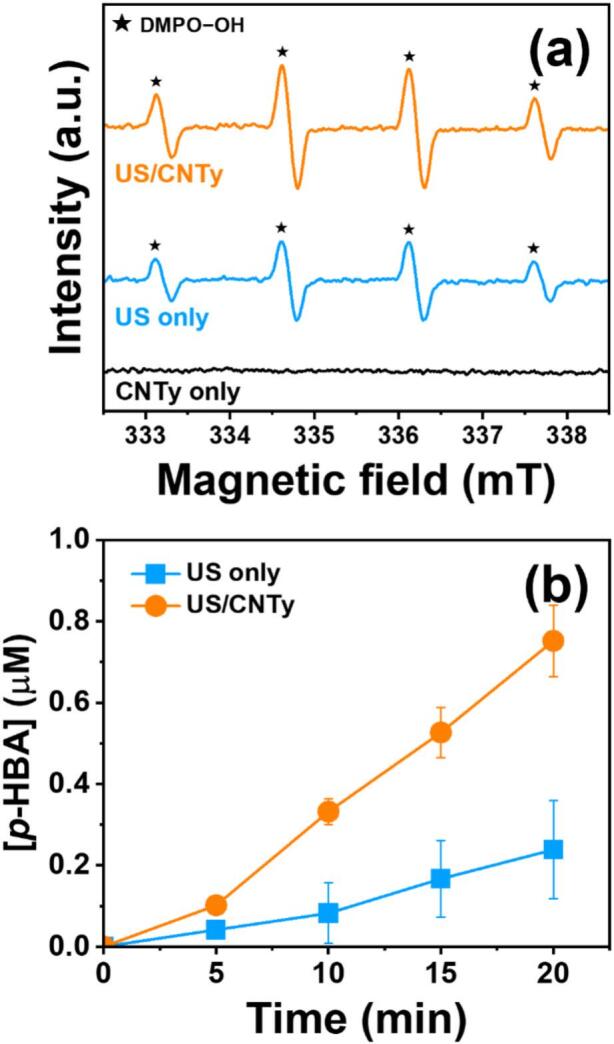
(a) EPR spectra showing DMPO–OH spin-trapping adduct signals under US only, CNTy only, and US/CNTy conditions and (b) quantification of cumulative ^•^OH concentration using BA as a probe in US only and US/CNTy systems ([DMPO]_0_ = 100 mM, [BA]_0_ = 10 mM, 100 W/L, [CNTy] = 200 mg/L, 5 mm, 20 min, 300 rpm, and 295 K).

Understanding the reaction mechanism is critical for optimizing US-assisted degradation systems and accurately interpreting the role of radical species in pollutant removal [56]. Accordingly, Fig. 7a shows the EPR spectra obtained by reacting DMPO as a spin-trapping agent for 5 min under US only and US/CNTy conditions. A characteristic quartet signal attributed to the DMPO-OH adduct is clearly observed in both systems, confirming ^•^OH generation via acoustic cavitation [57,58]. However, the signal intensity was relatively stronger in the US/CNTy system, indicating a significantly higher production of ^•^OH compared to US system. In contrast, no noticeable signal was observed in the CNTy only condition, demonstrating that the presence of US was essential for initiating radical formation. The enhanced DMPO-OH signal observed in the US/CNTy system highlights the synergistic role of CNTy in promoting ^•^OH generation during ultrasonic irradiation. This enhancement is likely due to the fact that CNTy provides favorable nucleation sites for cavitation bubbles, increasing the frequency and intensity of bubble collapse [59]. Fig. 7b illustrates the formation of *p*-HBA over time in the US alone and US/CNTy systems. After 20 min of treatment, the *p*-HBA concentration in the US/CNTy system reached approximately 0.75 µM, significantly exceeding the 0.24 µM observed in the US only condition [60]. The enhanced *p*-HBA formation in the US/CNTy system implies a higher amount of ^•^OH generation, which is thought to be due to the catalytic role of CNTy in promoting radical formation during acoustic cavitation. CNTy enhanced energy density, which promotes more frequent and intense cavitation events, thereby increasing ^•^OH generation. These results are consistent with the stronger DMPO–OH signal observed in the EPR spectra of the US/CNTy system.

**Fig. 8:**
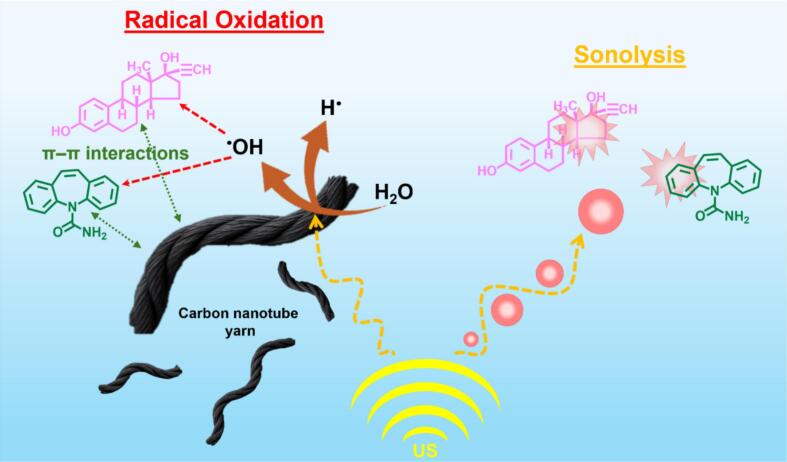
Schematic illustration of CBZ and EE2 degradation mechanism by US in the presence of CNTy.

The degradation of CBZ and EE2 in the US/CNTy system is promoted by the combined effect of acoustic cavitation and catalyst enhancement. High-frequency US (970 kHz) promotes rapid nucleation and collapse of microbubbles, generating ^•^OH radicals through intense cavitation [61]. Incorporating CNTy into the system further amplifies this process by providing favorable bubble nucleation sites on the surface, which increases both the frequency and intensity of cavitation [59]. In addition, CNTy promotes the formation of additional reactive species due to its high surface area and strong π-π interactions with aromatic contaminants and provides abundant active sites for contaminant adsorption [62]. This enhances the likelihood of effective collisions between contaminants and reactive radicals, and extends the interaction. Consequently, CBZ and EE2 underwent oxidative degradation via ^•^OH in both bulk solution and near the CNTy surface. This integrated mechanism resulted in a substantially improved degradation efficiency compared to the US only or CNTy only treatments. A schematic illustration of the proposed degradation mechanism is presented in Fig. 8.

## 4 Conclusions

This study successfully developed and validated a high-frequency US-assisted catalytic system incorporating CNTy for the efficient degradation of the recalcitrant pharmaceutical pollutants CBZ and EE2. The US/CNTy hybrid system demonstrated strong synergy by significantly intensifying acoustic cavitation and enhancing ^•^OH production, as evidenced by EPR spectroscopy. Notably, CNTy provide additional bubble nucleation sites, increasing the cavitation frequency and intensity, which improves pollutant adsorption and prolongs radical-pollutant interactions. Consequently, the degradation of kinetics improved substantially compared to those of the US only or CNTy only treatments. Under optimized conditions (100 mL solution, initial contaminant concentration 10.0 μM, pH 7.0, reaction time 40 min), the US/CNTy system achieved 90 % removal efficiency for CBZ and EE2 with calculated synergy indices of 1.39 and 1.73, respectively, confirming that CNTy significantly contributes to the ultrasonic catalytic process. Mechanistic investigations have highlighted that CNTy actively participate in radical chemistry and facilitate effective pollutant degradation pathways, rather than merely serving as passive support. These mechanistic insights establish a robust foundation for advanced sonocatalytic water-treatment technologies that target persistent micropollutants. Moreover, the US/CNTy system consistently exhibited a stable performance and excellent reusability when treating real surface water, underscoring its practical field applicability. Additionally, structural flexibility, formability, and durability of CNTs enable their versatile integration into diverse water treatment configurations. Future studies aimed at optimizing catalyst configuration and energy management are expected to further enhance its effectiveness and feasibility. Based on these promising findings, subsequent research will prioritize the validation of the optimal configurations and operational parameters for field scale CNTy based ultrasonic treatment systems.

## CRediT authorship contribution statement

**Jong-Soo Choi:** Writing – original draft, Data curation, Conceptualization. **Hak-Hyeon Kim:** Investigation, Formal analysis. **Lakshmi Prasanna Lingamdinne:** Software, Resources, Methodology. **Janardhan Reddy Koduru:** Software, Resources. **Yoon-Young Chang:** Methodology, Investigation. **Se Hoon Gihm:** Methodology, Funding acquisition. **Min Jang:** Resources, Investigation. **Chang Min Park:** Software, Resources. **Yeomin Yoon:** Writing – review & editing, Supervision.

## Declaration of competing interest

The authors declare that they have no known competing financial interests or personal relationships that could have appeared to influence the work reported in this paper.
