# Supplementary material for: Synergistic sonocatalytic degradation of carbamazepine and 17α-ethinylestradiol in the presence of carbon nanotube yarn
Source: Ultrason Sonochem. 2025 Nov 8;123:107670. doi: 10.1016/j.ultsonch.2025.107670 (PMC12648576; doi:10.1016/j.ultsonch.2025.107670)
Supplement: Supplementary Data 1 [file mmc1.docx]

**Supplementary Information**

**Synergistic sonocatalytic degradation of carbamazepine and**

**17α-ethinylestradiol in the presence of carbon nanotube yarn**

Jong-Soo Choi^a^, Hak-Hyeon Kim^a^, Lakshmi Prasanna Lingamdinne^b^,

Janardhan Reddy Koduru^b^, Yoon-Young Chang^b^, Se Hoon Gihm^c^, Min Jang^b^,

Chang Min Park^d^, Yeomin Yoon^a,^ *

*^a^Department of Environmental Science & Engineering, Ewha Womans University, Seoul 03760, Republic of Korea*

*^b^ Department of Environmental Engineering, Kwangwoon University, 447-1 Wolgye-dong Nowon-gu, Seoul,01897 Republic of Korea*

*^c^AweXome Ray Inc., Anyang, Gyeonggi-Do 14056, Republic of Korea*

*^d^Department of Environmental Engineering, Kyungpook National University, 80 Daehak-ro, Buk-gu, Daegu 41566, Republic of Korea*

**Corresponding author. E-mail: yoony@ewha.ac.kr (Y. Yoon)*

***Chemical reagents***

Ferrocene (C_10_H_10_Fe, >98%), thiophene (C_4_H_4_S, >99%), CBZ (C_15_H_12_N_2_O, >98%), EE2 (C_20_H_24_O_2_, >98%), and 5,5-dimethyl-1-pyrroline N-oxide (C_6_H_11_NO, DMPO, >98.0%) were purchased from Sigma-Aldrich (USA). Acetylene (C_2_H_2_, >99.9%), argon (Ar, >99.9%), and hydrogen (H_2_, >99.9%) gases were supplied by Donga Gas Co., Ltd. for CNTy synthesis (Republic of Korea). Phosphoric acid (H_3_PO_4_, >85%) and potassium phosphate salts, including monobasic (KH_2_PO_4_, >99.0%), dibasic (K_2_HPO_4_, >99.0%), and tribasic (K_3_PO_4_, >98.0%) salts, used for buffer preparation and pH control, were sourced from Fisher Chemicals (USA). Methanol (MeOH, >99.9%), acetonitrile (ACN, >99.9%), *tert*-butanol (*t*-BuOH, >99.9%), and benzoic acid (BA, >99.5%) were obtained from Samchun Chemicals, Co., Ltd (Republic of Korea). All aqueous solutions were prepared using deionized (DI, 18.2 MΩ) water, produced by a New Human Power III purification system (Human Corporation, Republic of Korea).

***Calorimetry Calculation***

1. Geometry

The ultrasonic generator bath (D 150 mm × W 100 mm × H 200 mm) with an ultrasonic generator mounted at the bottom and an ultrasonic power density of 100W/L. To estimate the proportion of energy delivered to the internal beaker reactor (diameter 70mm, capacity 250 mL), the ultrasonic irradiation power was calculated using Equation 1–4 based on the ratio of the beaker bottom area to the tank bottom area, yielding a result of 25.7 W/L.

$$Bottom area of the ultrasonic bath$$

$=150 mm\times100 mm=15,000 {mm}^{2}=150 {cm}^{2}$ (1)

$Bottom area of the beaker={(35 mm)}^{2}\times\pi\approx3,850 {mm}^{2}=38.5 {cm}^{2}$ (2)

$Area ratio=\frac{38.5 {cm}^{2}}{150 {cm}^{2}}=0.257$ (3)

$Applicable power density=0.257\times100 W/L=\boldsymbol{25.7}\boldsymbol{W}/\boldsymbol{L}$ (4)

1. Calorimetry

Ultrasonic output has been quantified using a calorimetric method widely employed in the ultrasonic field [1]. This method experimentally estimates the energy absorbed by the solution and converted into heat by measuring the slope of the solution temperature rise (ΔT/Δt) during the ultrasonic irradiation period (Table S4) [2]. The absorbed power P_abs_ is calculated as shown in Equation (5). The calculation of the relevant coefficients is as follows Equation 6–8. This approach assumes that all ultrasonic energy absorbed by the liquid is essentially converted into heat.

$P_{abs}={mc}_{p}\cdot\frac{\Delta T}{\Delta t}$ (5)

Heat capacity, ${mc}_{p}=0.1kg\times4186 J/{kg\cdot K}=418.6 J/K$ (6)

Chiller off: 298→327.1 K (ΔT=29.1 K) (7)

$P_{abs}=418.6 J/K\times\frac{29.1 K}{3600 s}\div0.1 L\approx\boldsymbol{33.8}\boldsymbol{W}/\boldsymbol{L}$ (8)

**References**

[1] T. Kikuchi, T. Uchida, Caloriametric method for measuring high ultrasonic power using water as a heating material, in: J. Phys. Conf. Ser., IOP Publishing 2011, pp. 012012.

[2] M. Plattes, C. Köhler, T. Gallé, Disequilibrium calorimetry for determination of ultrasonic power in sonochemistry, MethodsX 4 (2017) 274-278, <https://doi.org/10.1016/j.mex.2017.08.003>.

Table S1. Physicochemical properties of CBZ and EE2.

| Compound | Carbamazepine [CBZ] | 17α-Ethinylestradiol [EE2] |
| --- | --- | --- |
| Chemical formula | C_15_H_12_N_2_O | C_20_H_24_O_2_ |
| Molecular weight (g/mol) | 236.3 | 296.4 |
| p*K*_a_ | 13.9 | 10.4 |
| Log *K*_ow_ | 2.45 | 3.67 |
| Water solubility (mg/L at 25 ℃) | 17.7 | 11 |
| Chemical structure | 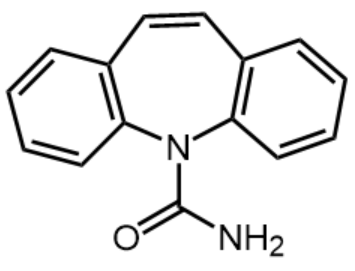 | 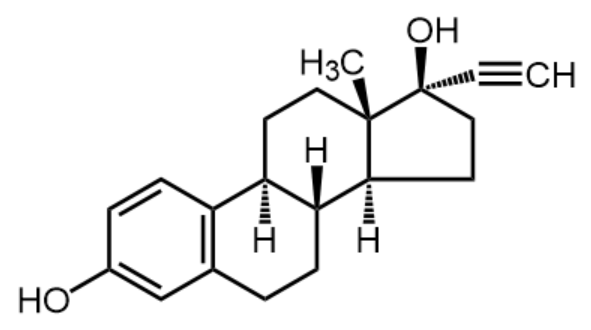 |

Table S2. Chromatographic conditions for the monitoring of contaminants by HPLC.

| Pollutant | Mobile phase (% v/v) | | | Flow in isocratic mode (mL/min) | Detector type | Detection wavelength (nm) |
| --- | --- | --- | --- | --- | --- | --- |
|  | MeOH | ACN | 10 mM phosphoric acid |  |  |  |
| CBZ | - | 50 | 50 | 0.8 | DAD | 210 |
| EE2 | 75 | - | 25 |  | FLD | 280 (Excitation)  310 (Emission) |

| **Parameter** | **Han River** |
| --- | --- |
| pH | 7.50 |
| TDS (mg/L) | 177 |
| EC (μS/cm) | 354 |
| TOC (mg/L) | 3.48 |
| Na^+^ (mg/L) | 12.92 |
| K^+^ (mg/L) | 4.09 |
| Mg^2+^ (mg/L) | 8.61 |
| Ca^2+^ (mg/L) | 35.70 |
| Cl^−^ (mg/L) | 12.60 |
| NO_3_^−^ (mg/L) | 8.09 |
| SO_4_^2−^ (mg/L) | 7.20 |

**Table S3.** Physicochemical properties of water sample collected from Jungnangcheon, a tributary of the Han River.

**Table S4.** Effect of temperature in ultrasonic treatment reactors with and without chillers.

| Time (min) | Chiller on (K) | Chiller off (K) |
| --- | --- | --- |
| 0 | 293.0 | 298.0 |
| 5 | 293.9 | 305.5 |
| 10 | 294.8 | 311.1 |
| 15 | 295.2 | 314.3 |
| 20 | 295.5 | 317.1 |
| 30 | 295.7 | 320.0 |
| 40 | 295.7 | 323.4 |
| 50 | 295.7 | 324.8 |
| 60 | 295.2 | 327.1 |


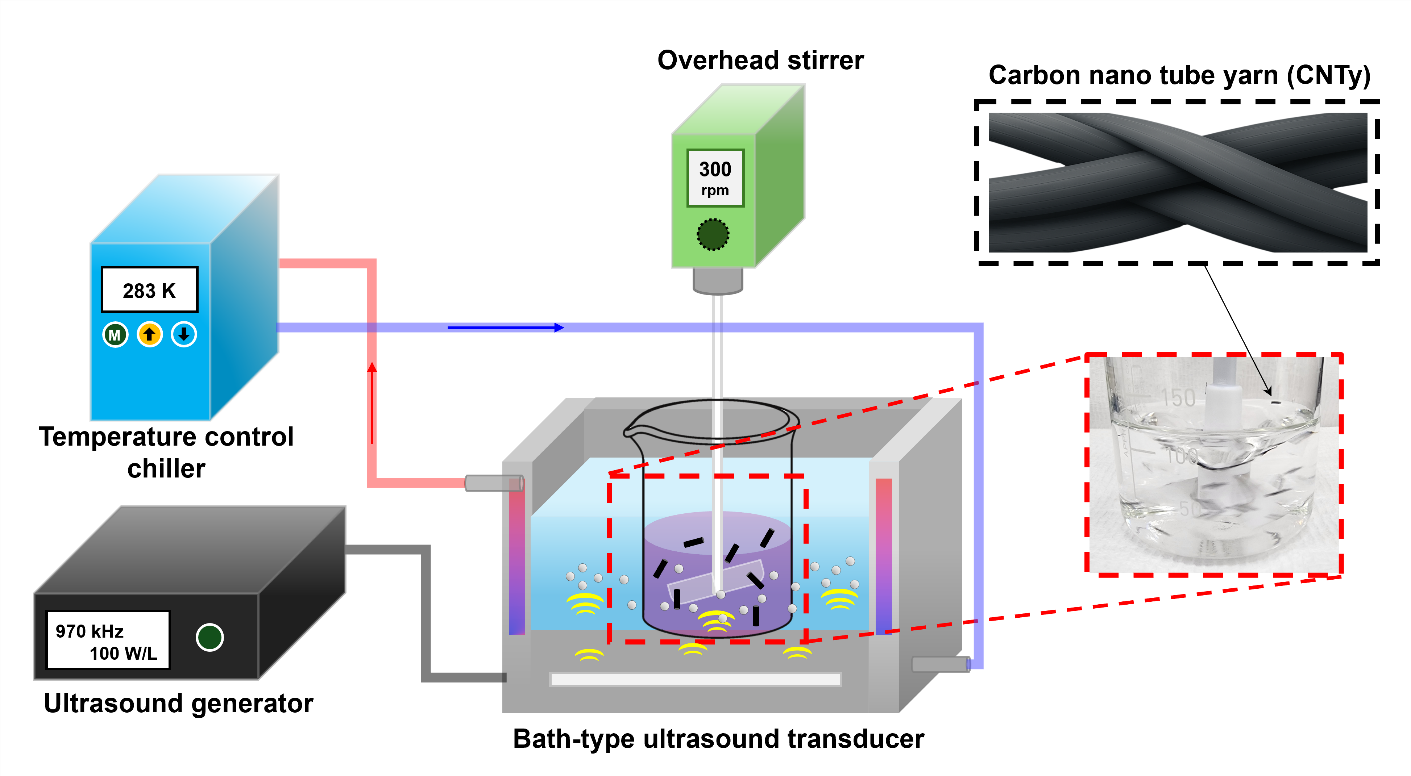


**Fig. S1.** Schematic illustration of the sonocatalytic system.


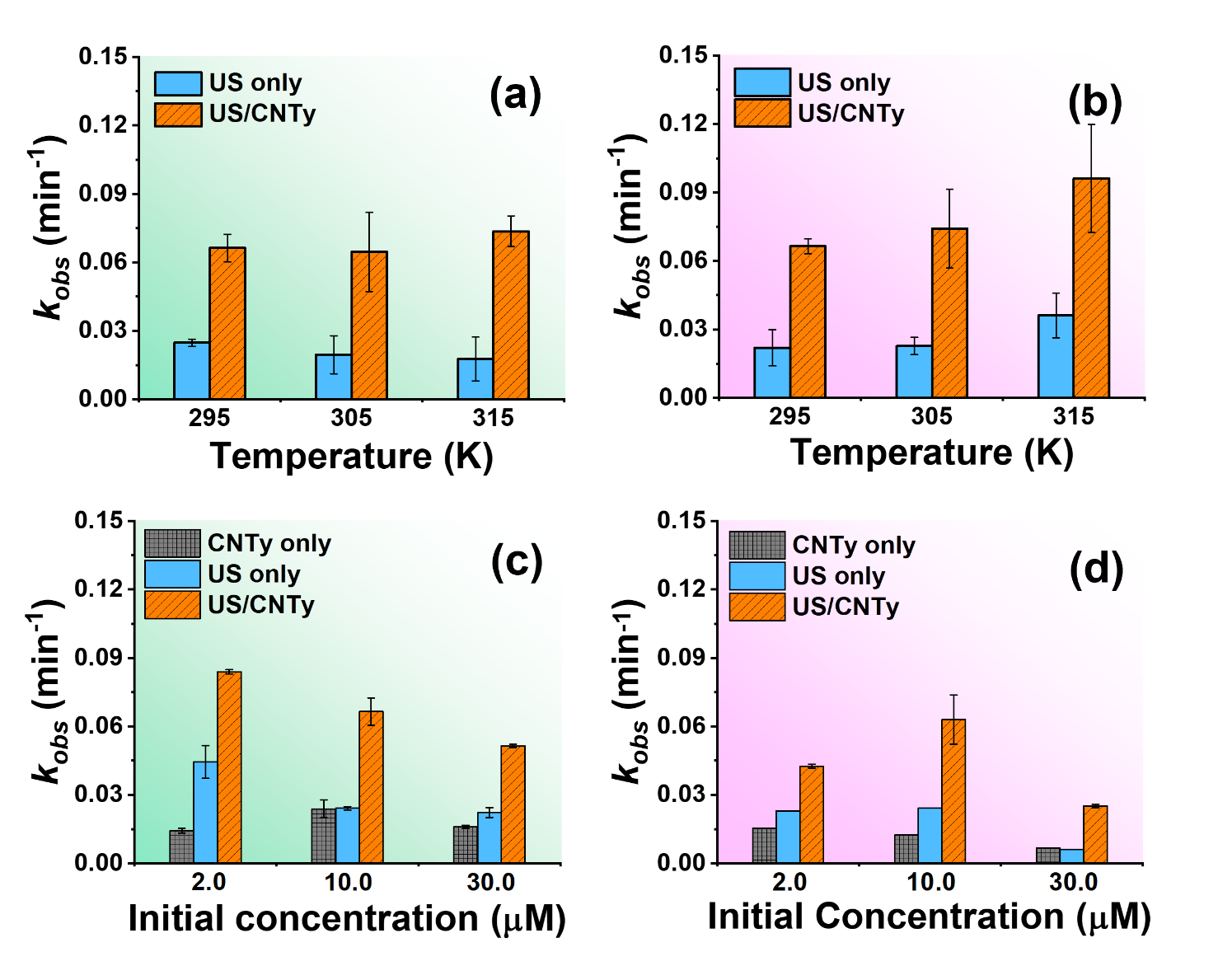


Fig. S2. Effects of (a and b) temperature and (c and d) initial concentration on the degradation rate for CBZ and EE2 degradation under CNTy only, US only, and US/CNTy processes ([CBZ]_0_ = [EE2]_0_ = 10 μM, pH 7.0, 100 W/L, [CNTy] = 200 mg/L, 5 mm, 20 min, 300 rpm, and 295 K).

**
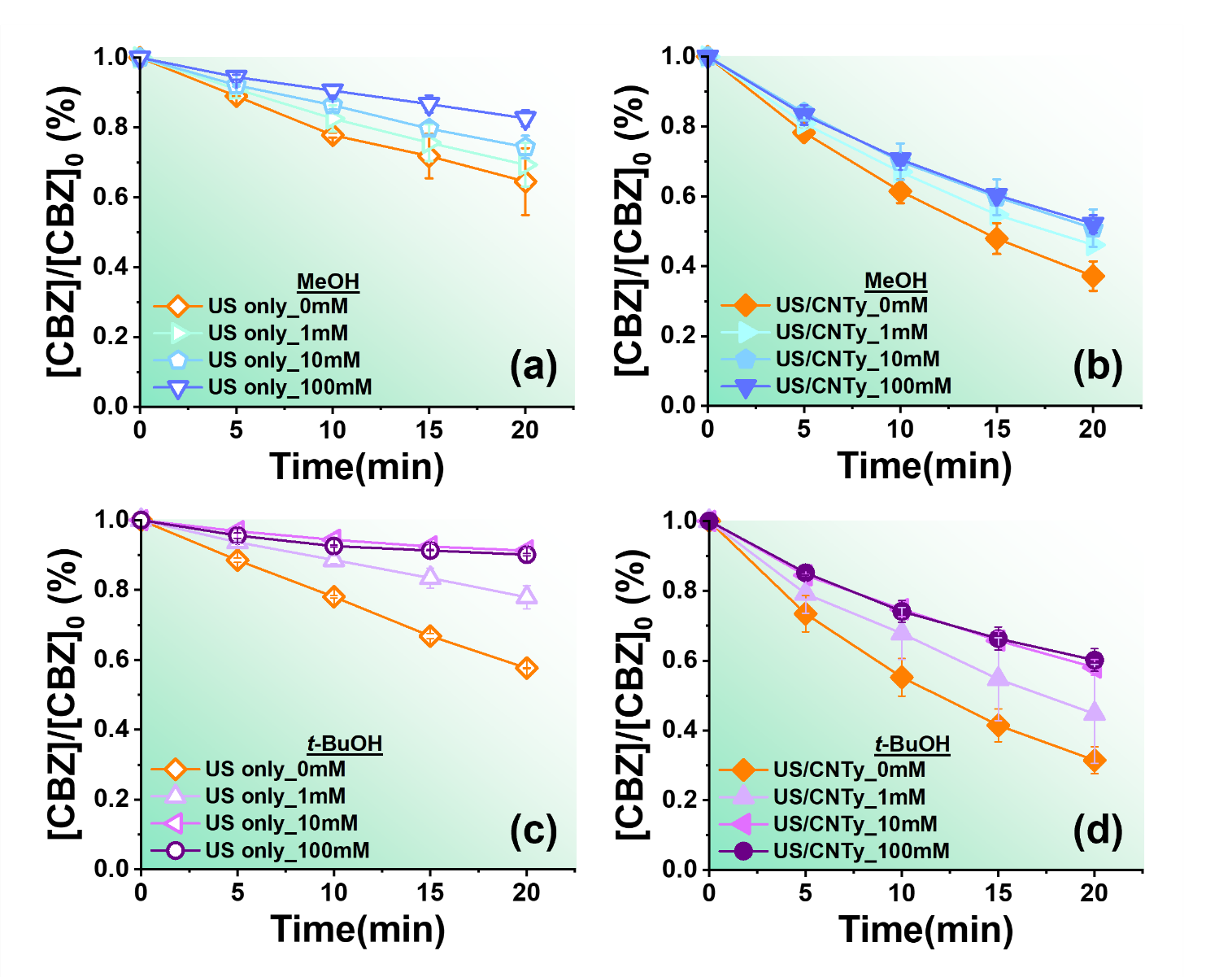
**

**Fig. S3.** Effect of varying concentrations of (a and b) MeOH and (c and d) *t*-BuOH on CBZ degradation under US only and US/CNTy conditions ([CBZ]_0_ = [EE2]_0_ = 10.0 μM, pH 7.0, 100 W/L, [CNTy] = 200 mg/L, 5 mm, 20 min, 300 rpm, 295 K, and [**s**cavenger] = 0 – 100 mM).

**
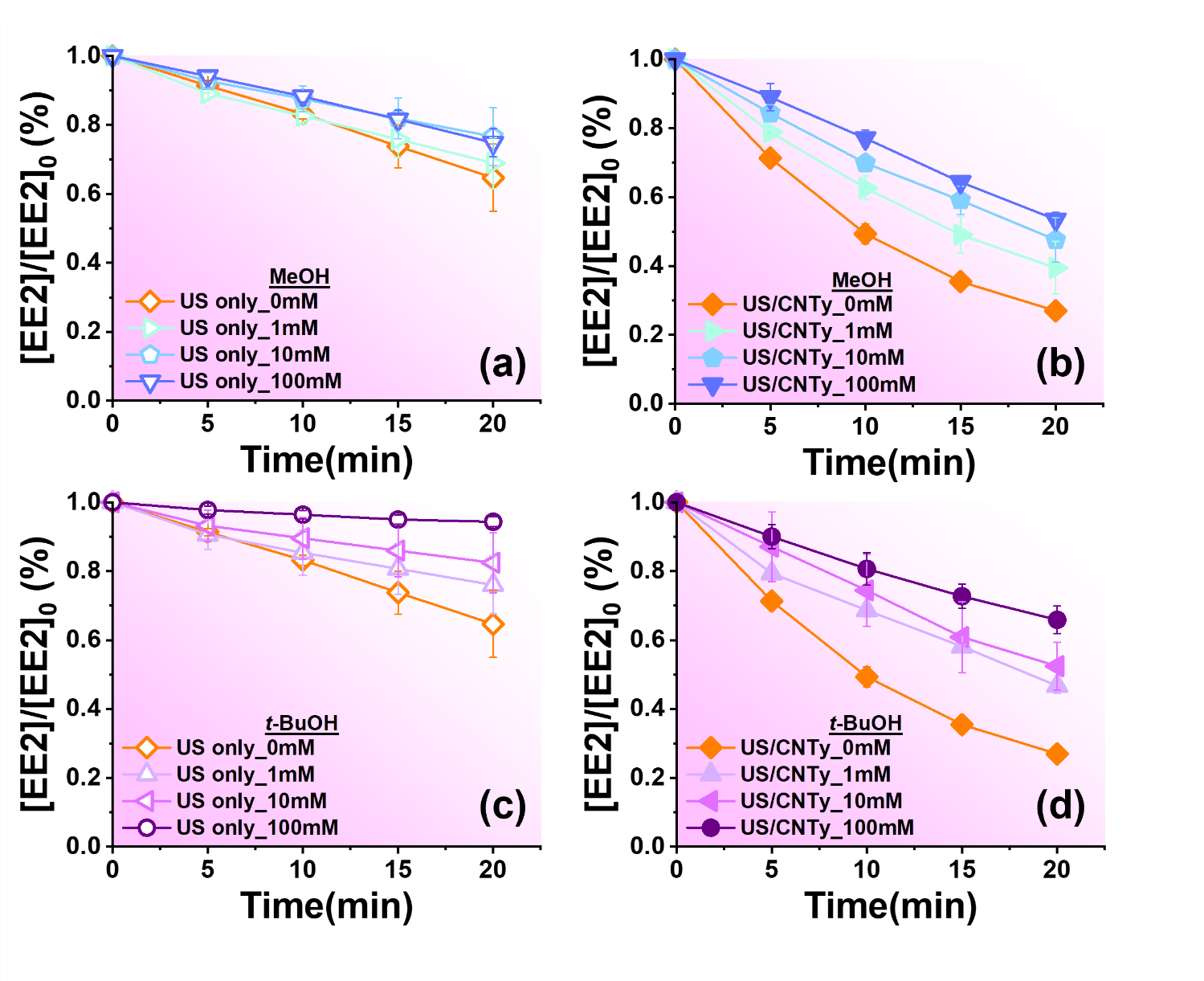
**

**Fig. S4.** Effect of varying concentrations of (a and b) MeOH and (c and d) *t*-BuOH on EE2 degradation under US only and US/CNTy system ([CBZ]_0_ = [EE2]_0_ = 10.0 μM, pH 7.0, 100 W/L, [CNTy] = 200 mg/L, 5 mm, 20 min, 300 rpm, 295 K, and [**s**cavenger] = 0 – 100 mM).

**
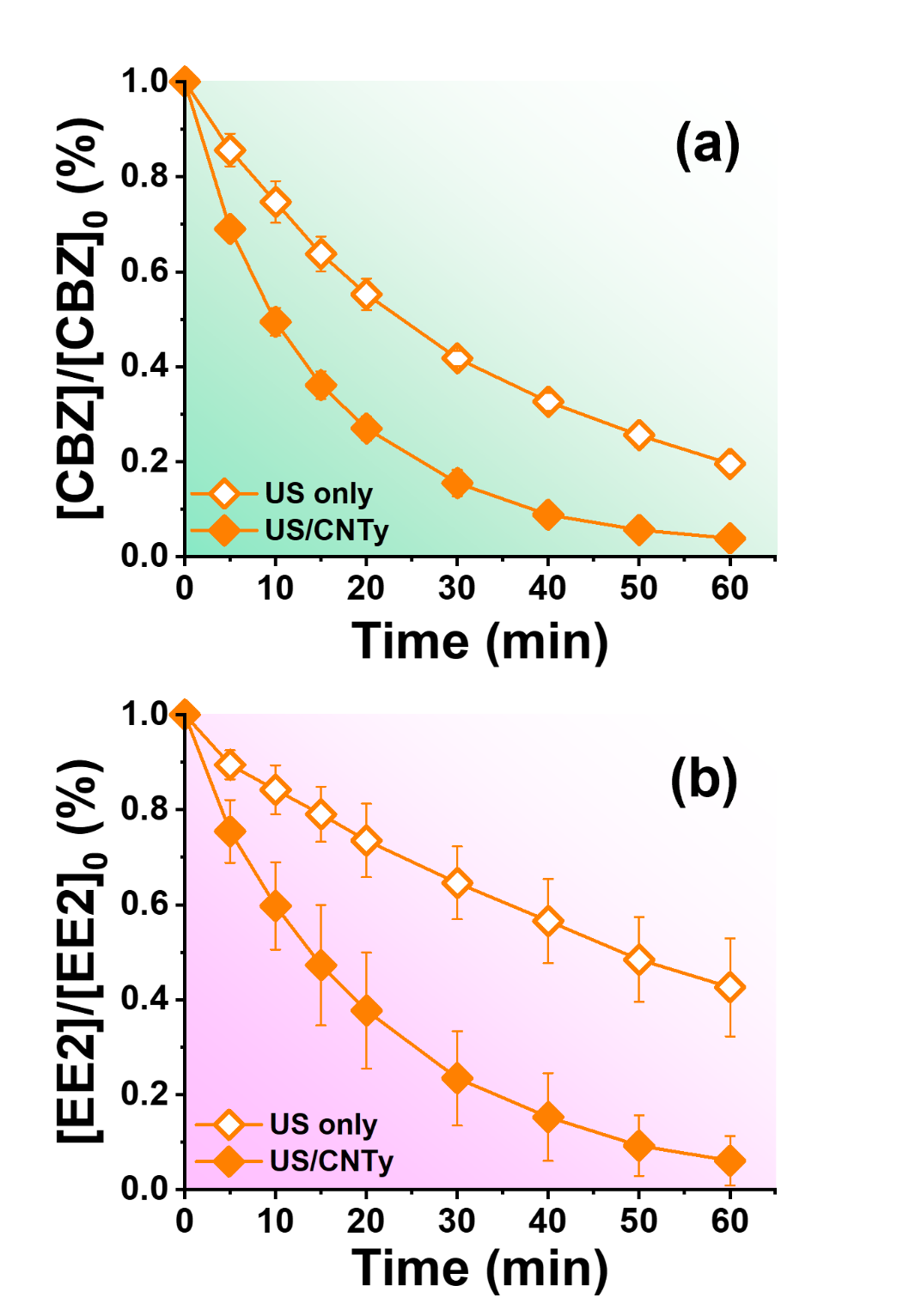
**

Fig. S5. Degradation performance of (a) CBZ and (b) EE2 under US only and US/CNTy treatment ([CBZ]_0_ = [EE2]_0_ = 10.0 μM, pH 7.0, 100 W/L, [CNTy] = 200 mg/L, 5 mm, 60 min, 300 rpm, and 295 K).
